# Evaluation und klinisches Management von interstitiellen Lungenanomalien: Positionspapier der Österreichischen Gesellschaft für Pneumologie (ÖGP) und Österreichischen Röntgengesellschaft (ÖRG) 2026

**DOI:** 10.1007/s00508-025-02687-4

**Published:** 2026-02-04

**Authors:** Tjaša Kamenski-Rathmanner, Georg Sterniste, David Lang, Kaveh Akbari, Franziska Arminger, Holger Flick, Barbara Gimpel, Maria-Anna Grabauer, Marie Therese Grasl, Mathis Hochrainer, Thomas Jaritz, Guangyu Shao, Helmut Prosch, Gerlig Widmann, Klaus Hackner

**Affiliations:** 1 Abteilung für Innere Medizin und Pneumologie, Klinikum Klagenfurt, Klagenfurt, Österreich; 2 Abteilung für Innere Medizin und Pneumologie, Klinik Floridsdorf, Karl Landsteiner Institut für Lungenforschung und Pneumologische Onkologie, Wien, Österreich; 3 Universitätsklinik für Innere Medizin 4 mit Schwerpunkt Pneumologie, Kepler Universitätsklinikum, Johannes Kepler Universität, Linz, Österreich; 4 Abteilung für Radiologie, Salzkammergut Klinikum Vöcklabruck, Vöcklabruck, Österreich; 5 Abteilung für Lungenkrankheiten, Klinikum Wels-Grieskirchen, Wels, Österreich; 6 Universitätsklinik für Innere Medizin, Klinische Abteilung für Pulmonologie, Medizinische Universität Graz, Graz, Österreich; 7 Abteilung für Innere Medizin und Pneumologie, Landeskrankenhaus Hochsteiermark, Leoben, Österreich; 8 Klinische Abteilung für Pneumologie, Universitätsklinikum Krems, Karl Landsteiner Privatuniversität für Gesundheitswissenschaften, Krems, Österreich; 9 Abteilung für Atemwegs- und Lungenkrankheiten, Standort Penzing der Klinik Ottakring, Ludwig Boltzmann Institut für Lungengesundheit, Wien, Österreich; 10 Universitätsklinik für Radiologie und Nuklearmedizin, Medizinische Universität Wien, Wien, Österreich; 11 Universitätsklinik für Radiologie, Medizinische Universität Innsbruck, Innsbruck, Österreich

**Keywords:** Lungenfibrose, CT Screening, CTD-ILD, Progressions- und Risikostratifizierung, Individuelles klinisches Follow-up, Pulmonary fibrosis, CT screening, CTD-ILD, Progression and risk stratification, Individualized clinical follow-up

## Abstract

Interstitielle Lungenanomalien (ILA) sind in der Computertomographie (CT) detektierte Befunde, die potenziell frühe Stadien interstitieller Lungenerkrankungen (ILD) widerspiegeln. Ihre Prävalenz variiert zwischen 3 und 10 % in der Allgemeinbevölkerung mit höheren Raten bei älteren Personen und RaucherInnen. ILA umfassen bilaterale und nicht-hypostasebedingte Milchglasveränderungen, retikuläre Anomalien, Traktionsbronchiektasien, Architekturstörungen und Honeycombing und betreffen mehr als 5 % einer Lungenzone. Das Progressionsrisiko zu ILD liegt je nach Subtyp und Risikofaktoren zwischen 20 und 80 %. Klinische Progressions- und Risikofaktoren sind Alter, Nikotinexposition, inhalative Noxen, thoraxchirurgische Eingriffe, pneumotoxische Therapien und abnorme Lungenfunktionsparameter. Radiologisch erhöhen fibrotische ILA mit subpleuraler und basaler Prädominanz sowie eine größere Ausdehnung das Progressionsrisiko signifikant. Das klinische Management basiert auf einer strukturierten Abklärung inklusive hochauflösender CT, Lungenfunktionsdiagnostik und Risikostratifizierung. Bei fehlenden Zeichen einer manifesten ILD empfiehlt sich ein individuelles Follow-up-Intervall von 6 bis 36 Monaten, abhängig vom Risikoprofil. Dieses Positionspapier formuliert praxisnahe Empfehlungen zum Umgang mit ILA und orientiert sich an aktuellen internationalen Leitlinien unter Berücksichtigung neuer Evidenz zu genetischen Risikofaktoren, progressionsassoziierten Bildgebungsmerkmalen und klinischen Prädiktoren. Ziel ist eine frühzeitige Identifikation von HochrisikopatientInnen und Vermeidung unnötiger diagnostischer oder therapeutischer Maßnahmen.

## Abstract (en)

Interstitial lung abnormalities (ILA) are findings detected on computed tomography (CT) that potentially reflect early stages of interstitial lung disease (ILD). Their prevalence ranges between 3–10% in the general population, with higher rates observed in older individuals and smokers. ILA include bilateral and nonhypostasis-related ground-glass opacities, reticular abnormalities, traction bronchiectasis, lung architectural distortion and honeycombing, affecting more than 5% of a lung zone. The risk of progression to ILD varies between 20–80%, depending on the ILA subtype and associated risk factors. Clinical progression and risk factors include advanced age, nicotine exposure, inhaled noxious substances, thoracic surgical procedures, pneumotoxic treatment and abnormal pulmonary function parameters. Radiologically, fibrotic ILA with subpleural and basal predominance as well as larger extent of lung involvement are significantly associated with increased risk of progression. The clinical management is based on a structured evaluation including high-resolution CT, lung function diagnostics and risk stratification. In the absence of signs of advanced fibrotic changes, individualized follow-up intervals ranging from 6–36 months are recommended, depending on the patient’s risk profile. This position paper provides practical recommendations for managing ILA, in line with current international guidelines, while considering new evidence on genetic risk factors, imaging features associated with progression and clinical predictors. The aim is an early identification of high-risk patients and avoidance of unnecessary diagnostic or therapeutic interventions.

## Definition und Prävalenz

Die Fleischner Society definiert in ihrem 2020 veröffentlichten Positionspapier interstitielle Lungenanomalien (ILA) als Zufallsbefunde in Thorax-Computertomographien (CT), die auf eine interstitielle Lungenerkrankung (ILD) hinweisen können und mehr als 5 % einer Lungenzone betreffen. Sie treten bei Personen auf, bei denen zunächst kein Verdacht auf eine solche Erkrankung besteht. Personen mit familiärer Fibrose oder rheumatischer Grunderkrankung sind von einer ILA-Diagnose ausgeschlossen [1]. Im Jahr 2025 veröffentlichte die American Thoracic Society (ATS) ein neues Statement, das sich in einem zentralen Punkt wesentlich von der bisherigen Definition von ILA unterscheidet: Das bisherige Ausschlusskriterium für HochrisikopatientInnen wurde aufgehoben. Somit können gemäß dieser Leitlinien ILA nun auch bei Personen mit einer familiären Lungenfibrose oder einer rheumatischen Grunderkrankung diagnostiziert werden, sofern keine manifeste ILD besteht [2]. Diese Änderung hat innerhalb der pneumologischen Fachgemeinschaft zu kontroversen Diskussionen geführt, insbesondere auch im Vergleich zur rezent publizierten EULAR/ERS-Leitlinie zu interstitiellen Lungenerkrankungen bei Kollagenosen (CTD-ILD), die in mehreren Punkten divergierende Positionen einnimmt [3]. Die spezifischen Unterschiede und Implikationen der Empfehlungen werden in diesem Positionspapier näher erläutert.

Die Prävalenz von ILA variiert und liegt in der Allgemeinbevölkerung zwischen 3 % und 10 %. Höhere Prävalenzen werden bei älteren Menschen und RaucherInnen beobachtet [1, 2, 4–9].

ILA beschreiben Lungenparenchymveränderungen einschließlich Milchglas- oder retikulärer Anomalien, Architekturstörungen, Traktionsbronchiektasien und/oder Honeycombing (Honigwabenmuster), die visuell > 5 % einer Lungenzone betreffen (Tab. 1 und 2). Die Veränderungen sind in der Regel bilateral und nicht lageabhängig, also nicht durch Schwerkraft und Körperlage im Rahmen der CT-Aufnahme beeinflusst. Der Schwellenwert von 5 % ist willkürlich gewählt und dient vor allem dazu, minimale, klinisch irrelevante Veränderungen auszuschließen, sowie dazu, die Vergleichbarkeit mit früheren Studien zu gewährleisten. Kritisch anzumerken ist jedoch, dass auch Läsionen mit einem Anteil von unter 5 % potenziell ein Progressionsrisiko bergen können [10]. Auch das ATS-Statement hält am Schwellenwert von 5 % fest, primär aufgrund des Fehlens validierter Alternativen [1, 2, 5]. Nichtemphysematöse Zysten, zentrilobuläre Noduli und Merkmale einer pleuroparenchymalen Fibroelastose können grundsätzlich vorhanden sein, tragen aber nicht zur Berechnung des Volumens der betroffenen Lungenanteile bei, das für die Definition einer ILA erforderlich ist [2].

**Tab. 1:** Interstitielle Lungenanomalien (ILA) – Definition nach ATS Clinical Statement 2025 und Fleischner Society Positionspapier 2021 (adaptiert & gekürzt)

ATS Clinical Statement	Fleischner Society Positionspapier
Bilaterale und nicht-hypostasebedingte Milchglasveränderungen, retikuläre Anomalien, Architekturstörungen, Traktionsbronchiektasien und/oder Honeycombing, die > 5 % einer Lungenzone betreffen*	Zufälliger Nachweis nicht schwerkraftabhängiger Auffälligkeiten einschließlich Milchglas- oder retikulärer Veränderungen, Architekturstörung Traktionsbronchiektasen, Honeycombing sowie nichtemphysematöser Zysten
Nichtemphysematöse Zysten, zentrilobuläre Noduli und Merkmale einer pleuroparenchymalen Fibroelastose können vorhanden sein, tragen aber nicht zum erforderlichen ILA-Volumen der betroffenen Lunge bei	Die Veränderungen betreffen mindestens 5 % einer Lungenzone*
In Hochrisikofällen (z. B. bei familiärer Lungenfibrose oder bekannten ILD-assoziierten genetischen Varianten) ist eine Bilateralität nicht erforderlich Die Notwendigkeit, dass Befunde zufällig sein müssen und Hochrisikopopulationen ausgeschlossen werden müssen, wurde bewusst aus der Definition gestrichen Leichte Anomalien, die ausschließlich in abhängigen Lokalisationen in der Rückenlage auftreten, sollten in der Bauchlage bestätigt werden	Bei Personen, bei denen keine interstitielle Lungenerkrankung vermutet wird *Was sind keine ILA (gekürzt):* präklinische interstitielle Auffälligkeiten, die im Rahmen eines Screenings bei Hochrisikopersonen identifiziert werden (z. B. bei PatientInnen mit rheumatoider Arthritis, Sklerodermie, berufsbedingter Exposition oder familiär auftretender interstitieller Lungenerkrankung)

*ILD* interstitielle Lungenerkrankung, *ILA* interstitielle Lungenanomalien

* Obere, mittlere und untere Lungenzonen werden durch die Ebenen des unteren Aortenbogens und der rechten unteren Lungenvene abgegrenzt, wodurch insgesamt 3 Zonen pro Seite entstehen

**Tab. 2:** Ergebnisse und Befunde, die nicht als interstitielle Lungenanomalien (ILA) betrachtet werden

Ausmaß	*Begrenzte Ausdehnung: *Begrenzte fokale Anomalie (z. B. fokale paraspinale „Reibungsfibrose“); einseitige Anomalie (außer bei Hochrisikopopulationen) *Ausgedehnte Veränderungen, die die Definition einer ILD erfüllen (Tab.* 3*)*
Befund, Muster, Ätiologie	Abhängige Lungenatelektase
	Unifokale oder multifokale lineare Vernarbung
	Nichtemphysematöse Zysten, zentrilobuläre Nodularität und/oder
	Merkmale einer pleuroparenchymalen Fibroelastose, ohne weitere CT-Befunde einer Lungenerkrankung
	Befunde einer Herzinsuffizienz
	Befunde einer Aspiration (z. B. fleckige Milchglaskonsolidierungen, Tree-in-bud-Zeichen)

*CT* Computertomographie, *ILD* Interstitielle Lungenerkrankung, *ILA* interstitielle Lungenanomalien

Das Positionspapier der Fleischner Society [1] identifizierte 3 Hauptkategorien von ILA: nichtsubpleurale ILA (d. h. ohne vorherrschende subpleurale Lokalisation), subpleurale nichtfibrotische ILA (d. h. mit vorherrschender subpleuraler Lokalisation, aber ohne Anzeichen einer Fibrose) und subpleurale fibrotische ILA (d. h. mit vorherrschender subpleuraler Lokalisation und Anzeichen einer Fibrose). Fibrose wurde durch das Vorhandensein einer Architekturstörung mit Traktionsbronchiektasien und/oder Honeycombing definiert und betrifft jene ILA, die die Kriterien für Ausmaß oder Muster einer ILD nicht erfüllen (Tab. 3).

**Tab. 3:** Definition der interstitiellen Lungenerkrankung für PatientInnen mit ILA gemäß dem ATS-Statement

Bei einer Person mit CT-Merkmalen von ILA muss mindestens eines der folgenden Kriterien erfüllt sein, um eine ILD* zu definieren
*Symptome:* Dyspnoe und/oder Husten jeglicher Art, die klinisch auf eine ILD zurückgeführt werden können
*Physiologie *(eine der folgenden): – Jede Verringerung in Lungenfunktionsparametern oder Diffusionsmarkern, die klinisch auf eine ILD zurückgeführt werden kann; – Zutreffen der Kriterien für eine progrediente pulmonale Fibrose (PPF) [12]
*Bildgebung *(eines der folgenden Kriterien in der Thorax-CT): – Fibrotische Anomalien (Honeycombing und/oder Retikulation mit Traktionsbronchiektasien), die visuell > 5 % des gesamten Lungenvolumens betreffen; – Progrediente fibrotische Anomalie in seriellen Thorax-CTs; – Vorhandensein eines typisch ausgeprägten fibrotischen ILD-Musters in der Thorax-CT (z. B. UIP/„probable UIP“, fibrotische HP oder fibrotische NSIP)
*Pathologie:* Bioptischer Nachweis eines typisch ausgeprägten fibrotischen ILD-Musters (z. B. UIP/„probable UIP“, fibrotische HP oder fibrotische NSIP)

*CT* Computertomographie, *HP* Hypersensitivitätspneumonitis, *ILA* interstitielle Lungenanomalien, *ILD* interstitielle Lungenerkrankung; *NSIP* nichtspezifische interstitielle Pneumonie; *UIP* „usual interstitial pneumonia“

* Zur Diagnose des spezifischen ILD-Typs können weitere diagnostische Untersuchungen erforderlich sein. Die Diagnose einer nichtfibrotischen ILD erfordert die Integration mehrerer Domänen

Zur Befundung von ILA sollte eine Thorax-CT mit dünnen Schichten (< 1,5 mm; hochauflösende [HR-]CT) erfolgen. Eine HR-CT wird ebenfalls empfohlen, wenn ein initialer Scan unvollständig sein sollte oder unklare Befunde bei größerer Schichtdicke liefert [1]. Eine ergänzende CT in Bauchlage kann eine sinnvolle Ergänzung sein, um lagebedingte Anomalien auszuschließen; und zeigt insbesondere bei nichtfibrotischen ILA eine bessere diagnostische Genauigkeit. Alleinige CTs in Rückenlage neigen dazu, das Ausmaß von ILA leicht zu überschätzen. Laut Fleischner Society ist eine CT in Bauchlage empfohlen, aber nicht zwingend notwendig [1, 11].

Rezente Studien haben eine erhebliche Variabilität der Prävalenz von ILA in verschiedenen Bevölkerungsgruppen gezeigt. Sie treten bei 4–17 % der Raucher und Lungenkrebs-Screening-Kohorten [13–16] sowie bei 3–10 % der bevölkerungsbasierten Kohorten [17–19] auf. Darüber hinaus sind ILA in asiatischen Bevölkerungen im Vergleich zu westlichen Bevölkerungen möglicherweise geringer [18, 20].

Die Variabilität der ILA-Prävalenz kann auf verschiedene Faktoren zurückgeführt werden, darunter Unterschiede in Kohortenmerkmalen wie Durchschnittsalter, genetische Prädispositionen, Umwelteinflüsse und Bildgebungsprotokolle. In einigen asiatischen Studien könnte der Einsatz einer Niedrigdosis-Thorax-CT zur Gesundheitsvorsorge oder zum Lungenkrebs-Screening zu den niedrigeren Prävalenzen beigetragen haben [20, 21]. Darüber hinaus ist der MUC5B-Promoter-Polymorphismus, ein anerkannter Risikofaktor für idiopathische Lungenfibrose (IPF), in der südkoreanischen Bevölkerung selten und könnte die regionalen Prävalenzunterschiede teilweise erklären [22]. Inkonsistente Definitionen von ILA erschweren die Prävalenzschätzungen zusätzlich, insbesondere bei älteren Studien und Berichten. Die Empfehlungen der Fleischner Society konnten zuletzt die Variabilität der berichteten Prävalenzraten reduzieren [1]. Unterschiede in den Bildgebungsverfahren und der Interpretation tragen jedoch zu der großen Bandbreite der Prävalenzschätzungen bei.

## Risikofaktoren für das Auftreten von interstitiellen Lungenanomalien

Klinische Risikofaktoren für das Auftreten von ILA lassen sich grob in demografische Merkmale, genetische Faktoren und inhalative Expositionen sowie das Vorhandensein einer Grunderkrankung, die bekanntermaßen mit der Entwicklung von ILD assoziiert ist, wie z. B. Kollagenosen, einteilen [1, 19, 23].

Unter den demografischen Merkmalen sind zunehmendes Alter und männliches Geschlecht von Bedeutung [15, 23]. Zunehmendes Alter ist einer der wichtigsten Risikofaktoren für das Auftreten von ILA [15, 17, 19]. In der Studie zur genetischen Epidemiologie der COPD (COPDGene) betrug die Prävalenz von ILA bei Personen unter 60 Jahren nur 4 % [24]. In der Framingham Heart Study betrug die Prävalenz von ILA bei Personen über 70 Jahren sogar 47 % [17]. In beiden Populationen war das Vorhandensein von ILA mit erhöhten Plasmaspiegeln des „growth differentiation factor“(GDF)-15 verbunden, einem Biomarker des Alters [17, 24]. Die Beobachtungen, dass das Fortschreiten von ILA bei älteren Menschen wahrscheinlicher ist und dass das erhöhte Risiko einer frühen Mortalität unabhängig vom Alter fortbesteht, legen nahe, dass ILA in dieser Population weiterhin klinisch relevant sind [16].

ILA wurden bei PatientInnen mit einer Lungenschädigung in der Anamnese (z. B. einer früheren Infektion) oder mit bekannter chronischer Lungenerkrankung beobachtet [25]. Es ist daher zu hinterfragen, ob anhaltende Milchglastrübungen und subtile subpleurale Retikulationen nach einer COVID-19-Pneumonie als ILA bezeichnet werden sollten. In der österreichischen, prospektiven, multizentrischen Nachsorgestudie (CovILD) wiesen PatientInnen mit schwerer COVID-19-Pneumonie in 54 % CT-Abnormalitäten nach einem Jahr auf [26]. In einer zweijährigen Nachuntersuchung zeigten 39 % der Überlebenden residuale Milchglastrübungen, die als Post-COVID-19-Lungenanomalien bezeichnet werden [27]. Die Autoren weisen darauf hin, dass diese Milchglastrübungen auch das Bild von ILA imitieren könnten, was sich auch in Unterschieden in der Assoziation mit genetischen Faktoren und den Langzeitergebnissen widerspiegelt. Unter den genetischen Faktoren ist der bisher stärkste gefundene Zusammenhang mit ILA ein Einzelnukleotid-Promotor-Polymorphismus (rs35705950) im Gen, das für MUC5B kodiert [7, 19, 28, 29]. Dieser Promotor-Polymorphismus ist bekanntermaßen mit IPF, familiärer Lungenfibrose und rheumatoider Arthritis-assoziierter ILD assoziiert [30, 31]. ILA sind mit einer verkürzten Telomerlänge assoziiert [32].

Unter den inhalativen Expositionen ist Tabakrauch der bedeutendste Risikofaktor [23, 29]. Auch andere inhalative Stoffe wie die berufliche Exposition gegenüber Dämpfen, Gasen, Stäuben, Rauch und verkehrsbedingter Luftverschmutzung spielen eine Rolle [33, 34].

## Progressionsfaktoren

**Abb. 1:**
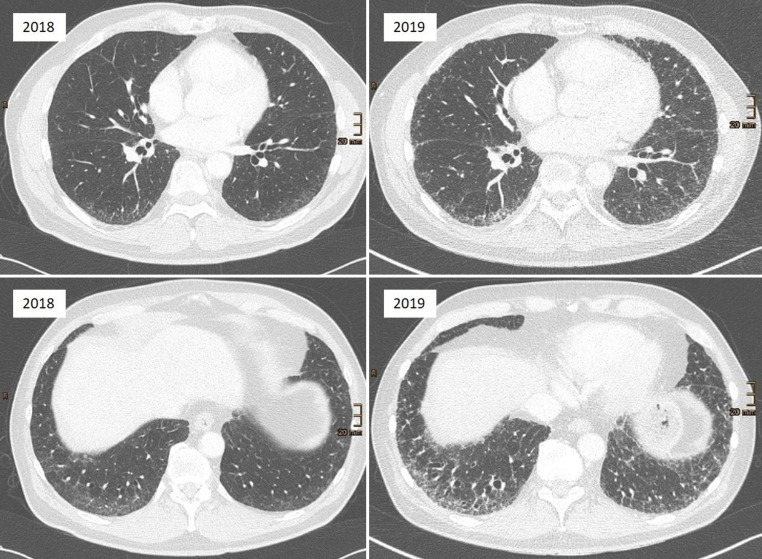
Beispiel für ein Voranschreiten von interstitiellen Lungenanomalien (ILA) zur interstitiellen Lungenerkrankung (ILD) binnen eines Jahres. 2018 zeigen sich in einem Kontrastmittel-unterstützten CT als Zufallsbefund basal betonte subpleurale Retikulationen und Milchglas sowie geringe posterobasale Traktionsbronchiektasien („subpleural fibrotic ILA“ nach Fleischner Society). 2019 zeigt sich die Progression zu einer ILD mit zunehmenden Retikulationen, Milchglas, Traktionsbronchiektasien und Lungenvolumenverminderung

Die Evidenzlage zu Progressionsfaktoren von ILA ist aufgrund der relativ jungen Definition dieser Entität bislang noch dünn. Dennoch erscheint angesichts der hohen Prävalenz von ILA eine differenzierte klinische Einschätzung des Progressionsrisikos essenziell [1, 7, 35]. Die bereits beschriebenen Risikofaktoren für ILA müssen nicht zwingend mit einer Progression assoziiert sein. Die berichteten Progressionsraten variieren erheblich zwischen den Studien und liegen – je nach Definition, Follow-up-Zeitraum und untersuchter Population – zwischen etwa 20 % und 80 % (Abb. 1) innerhalb des Beobachtungszeitraums [17, 35, 36].

Grundsätzlich lassen sich radiologische und klinische Progressionsmarker unterscheiden (Tab. 4). Die Fleischner Society identifizierte 3 radiologische Subtypen von ILA mit einem erhöhten Risiko für eine Krankheitsprogression [1]:

1. fibrotische ILA mit basaler und peripherer Prädominanz mit Honeycombing, entsprechend dem „usual interstitial pneumonia“(UIP)-Muster,
2. fibrotische ILA mit basaler und peripherer Prädominanz ohne Honeycombing, entsprechend dem „probable usual interstitial pneumonia“(probable UIP)-Muster,
3. nichtfibrotische ILA mit basaler und peripherer Prädominanz.

**Tab. 4:** Radiologische und klinische Risikofaktoren für eine Progression von interstitiellen Lungenanomalien

*Radiologische Risikofaktoren für Progression*
Fibrotische ILA (Honeycombing, Traktionsbronchiektasien, grobe Retikulationen) mit subpleuraler und basaler Dominanz
Ausgedehnter Befall in einer Lungenzone bzw. in der gesamten Lunge
Nichtfibrotische ILA mit subpleuraler und basaler Dominanz
*Klinische Risikofaktoren für Progression*
Nikotinkonsum und/oder Exposition gegenüber anderen inhalativen Noxen
Onkologische Therapien (Strahlentherapie, Chemo‑/Immuntherapie, thoraxchirurgische Eingriffe)
Abnorme oder grenzwertige Lungenfunktion (DLCO, FVC, TLC)
Höheres Alter

*ILA* interstitielle Lungenanomalien, *DLCO* Diffusionskapazität für Kohlenmonoxid, *FVC* forcierte Vitalkapazität, *TLC* totale Lungenkapazität

Die AGES-Reykjavik-Studie demonstrierte ein erhöhtes Progressionsrisiko bei subpleuralen Retikulationen und bei basalen Traktionsbronchiektasien sowie eine erhöhte Mortalität bei Vorliegen sowohl fibrotischer als auch nichtfibrotischer ILA. Das höchste Mortalitäts- und Progressionsrisiko wurde bei UIP und „probable UIP“-Mustern beobachtet [36, 37].

Eine im Jahr 2023 veröffentlichte Studie von Park et al. zeigte ergänzend, dass das Ausmaß der fibrotischen Veränderungen mit dem Progressionsrisiko korreliert – sowohl hinsichtlich der Ausdehnung pro Lungenzone als auch in Bezug auf die gesamte Lunge. Auch in dieser Analyse zeigte sich ein Hinweis auf eine erhöhte Mortalität bei PatientInnen mit ausgedehntem Befall durch fibrotische ILA. Nichtfibrotische Veränderungen waren in dieser Analyse hingegen mit keinem erhöhten Risiko assoziiert [30].

Die Beurteilung eines radiologischen Progresses gestaltet sich im klinischen Alltag häufig schwierig, da ILA typischerweise subtile Läsionen darstellen und ein geringer Progress visuell häufig schwer zu diagnostizieren ist. Quantitative, KI-gestützte CT-Analysen eröffnen das Potenzial, Progression objektiv und numerisch zu erfassen. Im klinischen Alltag stehen diese Verfahren jedoch derzeit noch nicht flächendeckend zur Verfügung, sodass die Einschätzung eines Progresses überwiegend visuell erfolgt.

Die Fleischner Society hat mehrere klinische Risikofaktoren benannt, die mit einem erhöhten Progressionsrisiko assoziiert sein können: Diese umfassen Nikotinabusus und weitere inhalative Expositionen sowie thoraxchirurgische Operationen oder onkologische Therapien wie eine Strahlentherapie oder die Anwendung potenziell pneumotoxischer Medikamente einschließlich bestimmter Chemotherapeutika sowie Checkpointinhibitoren. Auch grenzwertig pathologische Lungenfunktionsparameter – insbesondere eine reduzierte Diffusionskapazität (DLCO) oder eine leicht eingeschränkte forcierte Vitalkapazität (FVC) – können als potenzielle Prädiktoren für eine Progression gewertet werden (Tab. 4; [1]). Ebenso ist die Prävalenz von ILA bei RaucherInnen deutlich erhöht. Studien im Kontext fibrosierender Lungenerkrankungen zeigen zudem einen beschleunigten Lungenfunktionsverlust sowie eine erhöhte Mortalität bei Nikotinexposition. Vor diesem Hintergrund werden ein konsequenter Rauchstopp sowie die Vermeidung inhalativer Noxen dringend empfohlen [1, 2].

In der Framingham Heart Study war die Progression von ILA mit einem signifikant stärkeren Rückgang der forcierten Vitalkapazität assoziiert (−25 ml ± 11 ml; *p* = 0,03). Als einziger weiterer signifikanter Prädiktor wurde ein höheres Lebensalter identifiziert [17]. Eine Analyse der AGES-Reykjavik-Kohorte zeigte ein vergleichbares Ergebnis, wobei ein höheres Lebensalter mit einer verstärkten Progression von ILA assoziiert war [36]. Der Lungenfunktionsverlust als Progressionsfaktor konnte jedoch nicht in allen Studien konsistent nachgewiesen werden. Einige dieser Inkonsistenzen könnten durch Störfaktoren wie höheres Lebensalter und Nikotinanamnese erklärt werden – beides Variablen, die sowohl mit dem Auftreten von ILA als auch mit einer Abnahme der spirometrischen Messwerte assoziiert sind [35]. Aktuelle Empfehlungen berücksichtigen daher auch ein fortgeschrittenes Lebensalter sowie eine abnorme Lungenfunktion als unabhängige Risikofaktoren für die Progression fibrotischer Lungenerkrankungen [2]. Klinische Risikofaktoren haben eine wichtige Rolle in der Risikostratifizierung inne, jedoch ist der Umstand limitierend, dass die Evidenz bislang großteils auf retrospektiven Analysen großer Kohorten basiert, während prospektive Daten fehlen.

## Klinisches Management von ILA

**Abb. 2:**
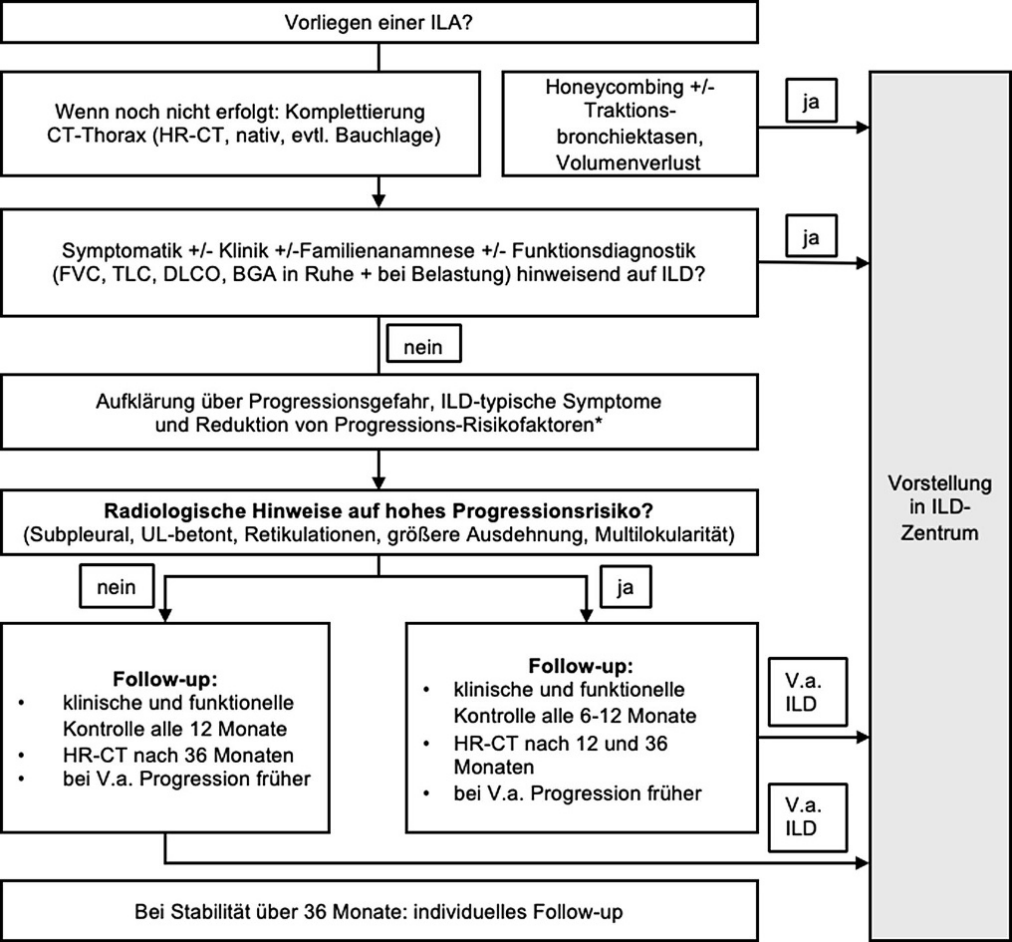
Klinisches Management bei Auftreten von interstitiellen Lungenanomalien. *Asterisk* In erster Linie Nikotinkonsum und weitere inhalative Noxen. *ILA* interstitielle Lungenanomalie, *ILD* interstitielle Lungenerkrankung, *CT* Computertomographie, *HR-CT* High-Resolution-Computertomographie, *FVC* forcierte Vitalkapazität, *TLC* totale Lungenkapazität, *DLCO* Diffusionskapazität, *BGA* Blutgasanalyse, *UL* Unterlappen

Die nachfolgenden Empfehlungen entstanden auf Basis einer kritischen Diskussion der aktuell verfügbaren Evidenzlage und definieren die aktuelle Position und Empfehlungen der Österreichischen Gesellschaft für Pneumologie (ÖGP) sowie der Österreichischen Röntgengesellschaft (ÖRG). Der entwickelte Algorithmus (Abb. 2) ist in seiner Validität nicht im Sinne einer prospektiven Studie überprüft worden, sondern stellt eine Zusammenfassung von ExpertInnenmeinungen und evidenzbasierten Empfehlungen dar.

### Vorliegen einer ILA

Der erste entscheidende Schritt im diagnostischen Algorithmus (Abb. 2) besteht in der klaren Abgrenzung zwischen PatientInnen mit manifester ILD und solchen ohne signifikante Erkrankung (ILA).

### Komplettierung der CT

ILA können gelegentlich auch inzidentell in bildgebenden Verfahren erkannt werden, die nicht primär zur Beurteilung des Thorax durchgeführt wurden – etwa in den basalen Lungenabschnitten bei Abdomen-CTs oder kardialen CT-Untersuchungen. In solchen Fällen sollte zur weiterführenden Abklärung eine vollständige CT des Thorax erfolgen, in Zweifelsfällen auch eine HR-CT in Bauchlage.

### Vorliegen von manifesten Fibrosezeichen

Bei radiologischen Hinweisen auf eine manifeste fibrosierende Lungenerkrankung – insbesondere bei Nachweis von Volumenverlust, Honeycombing oder Traktionsbronchiektasien – sollte eine zeitnahe Vorstellung in einem spezialisierten ILD-Zentrum erfolgen, auch wenn es sich um Zufallsbefunde handelt.

### Weitere Abklärung

Auch wenn keine signifikante ILD vorliegt, ist eine umfassende klinische Abklärung erforderlich. Diese sollte eine ausführliche Anamnese (einschließlich Familienanamnese, rheumatologischer Anamnese, beruflicher und umweltbezogener Expositionen, Risikofaktoren sowie respiratorischer Symptome) und eine körperliche Untersuchung umfassen. Darüber hinaus wird eine Lungenfunktionsprüfung empfohlen – mindestens mittels Spirometrie zur Messung der FVC; idealerweise ergänzt durch Bodyplethysmographie – mit Messung der totalen Lungenkapazität (TLC) und Messung der Diffusionskapazität (DLCO), sofern verfügbar [1, 2].

Im Falle einer lungenfunktionellen Einschränkung oder respiratorischen Symptomatik muss differenzialdiagnostisch jedenfalls erwogen werden, ob alternative Ursachen – etwa obstruktive Lungenerkrankungen oder kardiale Ursachen – verantwortlich sein können. Ergibt sich aus der Befundkonstellation der Verdacht auf eine ILD, ist ebenfalls eine Überweisung an ein ILD-Zentrum zu veranlassen. Das ATS 2025 Statement betont, dass anamnestisch jedenfalls Husten und (Belastungs‑)Dyspnoe erhoben werden sollten. Observationsstudien haben gezeigt, dass eine respiratorische Symptomatik mit schlechterem Outcome vergesellschaftet ist. Werden die Symptome auf die vorliegenden interstitiellen Veränderungen zurückgeführt, spricht dies für die Diagnose einer ILD [2, 10, 38].

Genetische Faktoren wie Mutationen im *MUC5B*-Gen oder Telomeropathien sind als Risikofaktoren für die Progression fibrotischer Erkrankungen bekannt. Derzeit beschränken sich entsprechende genetische Analysen jedoch auf den Rahmen wissenschaftlicher Studien. Eine routinemäßige Testung abseits von klar familiär gehäuften ILD-Fällen wird im klinischen Alltag aktuell nicht empfohlen, da sie mit erheblichem Aufwand sowie hohen Kosten für das Gesundheitssystem verbunden wäre. Auch eine histologische Sicherung der interstitiellen Veränderungen wird aufgrund des ungünstigen Nutzen-Risiko-Profils nicht empfohlen [10].

### Follow-up

Sofern tatsächlich ILA vorliegen, sollte im nächsten Schritt eine individuelle Risikostratifizierung erfolgen, um ein adäquates Follow-up zu gewährleisten. Hierbei ist zu berücksichtigen, dass bislang nur limitierte Evidenz hinsichtlich optimaler Nachsorgeintervalle – sowohl radiologisch als auch funktionell – vorliegt. Eine Risikoreduktion ist, wenn möglich, immer indiziert.

Die Fleischner Society empfiehlt in ihrem Positionspapier lungenfunktionelle Kontrollen in Intervallen von 3 bis 12 Monaten. Bildgebende Verlaufskontrollen mittels Computertomographie sollten alle 12 bis 24 Monate erfolgen. Dabei ist besonders zu betonen, dass bei klinischem oder radiologischem Verdacht auf eine Krankheitsprogression eine frühzeitige Wiedervorstellung sowie erneute Beurteilung notwendig ist – es sollte nicht starr an den empfohlenen Intervallen festgehalten werden [2]. Anhand der genannten klinischen und radiologischen Risikofaktoren kann eine Einteilung der PatientInnen in Gruppen mit „niedrigem Risiko“ sowie „erhöhtem Risiko“ erfolgen, um eine strukturierte und individualisierte Verlaufsbeobachtung zu ermöglichen. Das ATS-Statement empfiehlt für HochrisikopatientInnen vergleichbare Intervalle: Lungenfunktionstests alle 6 bis 12 Monate sowie eine CT-Kontrolle nach 12 Monaten. Für PatientInnen mit geringem Risiko erscheinen hingegen auch längere Beobachtungszeiträume von 2 bis 3 Jahren vertretbar [2]. In Anlehnung daran empfehlen wir daher ein vergleichbares Vorgehen.

Eine bislang offene Fragestellung betrifft die Dauer der Nachbeobachtung von ILA. Eine lebenslange Verlaufskontrolle, insbesondere unter Einbeziehung wiederholter CT-Untersuchungen, stellt sowohl für die PatientInnen als auch für das Gesundheitssystem eine erhebliche Belastung dar. Daten der Park-Studie zeigten, dass die mediane Zeit bis zur Progression einer ILA bei 3,2 Jahren lag. Die Entwicklung eines UIP-Musters benötigte hingegen deutlich mehr Zeit, mit einem Median von 11,8 Jahren für den Übergang von ILA zu UIP [30]. Bei stabilen Verläufen über 36 Monate hinaus sollte somit ein individuelles weiteres Follow-up gewählt werden, abhängig von den vorhandenen Progressionsrisikofaktoren, der Präsentation der ILA und dem Alter.

### Lungenkrebsscreening

Angesichts der hohen Mortalität des Lungenkarzinoms ist die Einführung eines strukturierten Lungenkrebsscreenings aktuell Gegenstand gesundheitspolitischer Debatten. Während einige Länder bereits, internationalen Empfehlungen folgend, Programme etabliert haben, zeigen sich andere – insbesondere aus gesundheitsökonomischen Gründen – noch zurückhaltend. Eine zentrale Herausforderung besteht in der Häufigkeit inzidenteller Befunde, die über die reine Tumordetektion hinausgehen, etwa ILA. Diese können für Betroffene sowohl Nutzen als auch Schaden bedeuten und verursachen zusätzliche Kosten [2, 39].

Ein erhöhtes biologisches Alter sowie Nikotinabusus gelten als etablierte Risikofaktoren sowohl für die Entstehung von Lungenkarzinomen als auch für ILA und viele ILD. Daher definieren die meisten Lungenkrebsscreening-Programme Einschlusskriterien, typischerweise einen Altersbereich zwischen 50 und 75 Jahren sowie eine positive Raucheranamnese. Ein Benefit solcher Screeninguntersuchung bezüglich der Lungenkrebsmortalität konnte in mehreren Studien gezeigt werden. In Europa wurden bzw. werden solche Programme bereits in Ländern wie den Niederlanden, Belgien, Frankreich, Polen, Kroatien und Deutschland etabliert [40–43].

Wichtig erscheint in diesem Zusammenhang, dass bei Durchführung des Lungenkrebsscreenings auch eine systematische Erfassung und Dokumentation von ILA erfolgen sollte, damit diese nicht „vergessen“ oder „übersehen“ werden.

Das Follow-up von ILA, die im Rahmen des Lungenkrebsscreenings detektiert werden, unterscheidet sich in Teilen vom standardisierten Vorgehen außerhalb des Screenings. Der wesentliche Unterschied liegt in der Frequenz der bildgebenden Kontrollen: Während im Lungenkrebsscreening standardmäßig jährliche CT-Untersuchungen erfolgen, richtet sich die Kontrollfrequenz bei ILA primär nach dem individuellen Progressionsrisiko – sowohl radiologisch als auch klinisch. Werden ILA im Rahmen des Lungenkarzinomscreenings identifiziert, erfolgt somit automatisch eine jährliche bildgebende Verlaufskontrolle. Bei Hinweisen auf eine Progression sollte eine zeitnahe Vorstellung in einem spezialisierten ILD-Zentrum erfolgen. Zusätzlich werden klinisch-funktionelle Verlaufsuntersuchungen in Intervallen von 6 bis 12 Monaten empfohlen.

### Familiäre Fibrose

Das ATS-Statement 2025 empfiehlt ein Thorax-CT-Screening auf ILA/ILD bei Erwachsenen > 50 Jahren mit einem Verwandten ersten Grades und familiärer pulmonaler Fibrose (FPF). FPF ist definiert durch ≥ 2 genetisch verwandte Angehörige ersten oder zweiten Grades mit fibrotischer ILD. Liegt nur ein betroffener Verwandter vor, wird keine Empfehlung ausgesprochen [2].

### Diskussion – CTD-ILD

Da mittlerweile mehrere Leitlinien (u. a. das Fleischner Society ILA-Positionspapier, das ATS-Clinical Statement zu ILA sowie die EULAR/ERS-Leitlinie zu CTD-ILD) existieren, ergeben sich im Kontext von interstitiellen Veränderungen im Rahmen von CTD teils widersprüchliche Empfehlungen [1, 2, 44].

Das Fleischner-Positionspapier schließt PatientInnen mit bekannten Hochrisikokonstellationen wie CTD oder familiärer Lungenfibrose explizit von der Definition der ILA aus. Im Gegensatz dazu verfolgt das ATS-Statement einen weiter gefassten Ansatz und schließt diese Gruppen bewusst ein – die amerikanische Leitlinie argumentiert, dass die Einbeziehung von Hochrisikopopulationen in die ILA-Definition eine bessere Grundlage für Empfehlungen zur Bewertung, Überwachung und zum Management dieser PatientInnengruppen schafft. Zudem sei es praktischer, eine breitere und einheitliche Definition von ILA anzuwenden, die auf verschiedene klinische Kontexte übertragbar ist.

Gleichzeitig ist jedoch gut belegt, dass Lungenbeteiligungen bei CTD häufig auftreten und entscheidend für Prognose und Mortalität sind. Eine zu weit gefasste Definition von ILA birgt das Risiko, klinisch relevante interstitielle Veränderungen bei CTD-PatientInnen als unspezifisch zu bewerten und dadurch zu unterschätzen oder inadäquat zu behandeln. Studien zeigen, dass selbst initial geringe oder „subklinische“ interstitielle Veränderungen („ILA“) mit einer ähnlichen Krankheitsprogression wie manifeste ILD einhergehen – bereits minimale fibrotische Veränderungen sind mit erhöhter Mortalität assoziiert [45]. Eine präzise Abgrenzung zwischen ILA im engeren Sinn und CTD-assoziierter ILD bleibt daher essenziell, um eine adäquate Risikostratifizierung und Therapieplanung zu gewährleisten.

Ein weiterer Unterschied zwischen den Empfehlungen betrifft das Screening auf interstitielle Veränderungen mittels HR-CT bei CTD. Das ATS-Statement (2025) empfiehlt eine HR-CT als Basisuntersuchung bei Erwachsenen mit erhöhtem ILD-Risiko (PICO Question 2), darunter RA, SSc, Polymyositis/Dermatomyositis, Antisynthetasesyndrom, MCTD, Sjögren-Syndrom und Overlap-Syndrome. Im Gegensatz dazu verfolgen die EULAR/ERS-Leitlinien grundsätzlich einen risikoadaptierten Ansatz für das HR-CT-Screening. Eine Ausnahme bilden jedoch PatientInnen mit SSc und/oder MCTD, bei denen aufgrund des hohen Prävalenz- und Progressionsrisikos eine HR-CT unabhängig von weiteren Risikofaktoren empfohlen wird. Darüber hinaus wird in den EULAR/ERS-Empfehlungen die Stärke der Evidenz graduell differenziert (starke vs. bedingte Empfehlung vs. klinische Praxis).

Insgesamt zeigen sich insbesondere im Umgang mit interstitiellen Veränderungen bei Kollagenosen (CTD) gemäß den aktuellen Leitlinien uneinheitliche und teils widersprüchliche Empfehlungen, die mehrere offene Fragen hinterlassen. Nach Einschätzung der AutorInnen dieses Positionspapiers als Stellvertreter ihrer Fachgesellschaften sollen auch geringe interstitielle Veränderungen im Kontext von CTD stets differenziert, multidisziplinär und im besten Fall an erfahrenen Zentren beurteilt und keinesfalls pauschal als ILA eingeordnet werden. Angesichts der bekannten prognostischen Bedeutung einer pulmonalen Beteiligung bei CTD ist eine präzise diagnostische Zuordnung entscheidend, um eine adäquate Risikostratifizierung und Therapieplanung zu gewährleisten [1–3]. Solange neue Studiendaten diese Herangehensweise nicht sicher untermauern, empfehlen die AutorInnen daher, den Begriff ILA im Kontext von Hochrisikopopulationen nicht zu verwenden, um keinerlei Unterdiagnostik zulasten der Prognose zuzulassen.

## Conclusio

ILA sind häufige Zufallsbefunde in der CT, die ein frühes Stadium einer ILD anzeigen können und auch unabhängig davon mit einer erhöhten Sterblichkeit vergesellschaftet sind. Das Progressionsrisiko zur ILD liegt abhängig vom radiologischen Subtyp und vorliegenden Risikofaktoren zwischen 20 und 80 %, wobei Alter, Rauchen und fibrotische Veränderungen besonders relevant sind. Das klinische Management umfasst strukturierte Abklärung, Risikostratifizierung und ein individuell daran angepasstes Follow-up. Ziel ist es, HochrisikopatientInnen frühzeitig zu erkennen und zu behandeln, aber gleichzeitig unnötige Diagnostik und Therapie zu vermeiden.
